# Network embedding aided vaccine skepticism detection

**DOI:** 10.1007/s41109-023-00534-x

**Published:** 2023-02-16

**Authors:** Ferenc Béres, Tamás Vilmos Michaletzky, Rita Csoma, András A. Benczúr

**Affiliations:** 1 ELKH Institute for Computer Science and Control (SZTAKI), Kende u. 13-17, Budapest, 1111 Hungary; 2 Eötvös Loránd University, Pázmány Péter s. 1, Budapest, 1117 Hungary

## Abstract

We investigate automatic methods to assess COVID vaccination views in Twitter content. Vaccine skepticism has been a controversial topic of long history that has become more important than ever with the COVID-19 pandemic. Our main goal is to demonstrate the importance of network effects in detecting vaccination skeptic content. Towards this end, we collected and manually labeled vaccination-related Twitter content in the first half of 2021. Our experiments confirm that the network carries information that can be exploited to improve the accuracy of classifying attitudes towards vaccination over content classification as baseline. We evaluate a variety of network embedding algorithms, which we combine with text embedding to obtain classifiers for vaccination skeptic content. In our experiments, by using Walklets, we improve the AUC of the best classifier with no network information by. We publicly release our labels, Tweet IDs and source codes on GitHub.

## Introduction

Vaccine skepticism, a controversial topic of long-past history, became more important than ever with the COVID-19 pandemic. Studies of social networks show that opposition to vaccines is small but far-reaching and could undermine COVID-19 vaccination efforts (Ball 2020). Only a year after the first international cases were registered, multiple vaccines were developed and passed clinical testing. Besides the challenges of development, testing and logistics, another factor in the fight against the pandemic are people who are hesitant to get vaccinated or even state that they will refuse any vaccine offered to them.

We focus on two groups of people commonly referred to as (a) pro-vaxxer, those who support vaccinating people (b) vax-skeptic, those who question vaccine efficacy or the need for general vaccination against COVID-19. It is very difficult to tell exactly how many people share these views. It is even more challenging to understand the mechanisms of vax-skeptic opinion spreading, which may be related to the positive correlation (McMullan et al. 2019) between health anxiety and the consumption of online health information, which is often inaccurate, misleading, or incomplete (Eysenbach et al. 2002).

We develop techniques that can efficiently differentiate between pro-vaxxer and vax-skeptic content. As a core solution and baseline method, we apply text classification based on embedding words of the text content into vector spaces (Mikolov et al. 2013). Automated categorization or classification of text into predefined categories has been an active area of research for decades (Sebastiani 2002). The dominant approach to this problem is based on machine learning from a set of manually labeled text. Embedding words into a vector space by relying on attention neural networks recently emerged as state-of-the-art (Devlin et al. 2018), with improved variants pre-trained on huge text collections (Brown et al. 2020) appearing frequently. Given a vector representation of each word, classification can be performed by any method suitable for numeric attributes, such as logistic regression or neural networks.

Despite the progress in text classification, inferring opinion and emotion remains particularly challenging for short Twitter messages. Text classification methods largely rely on word co-occurrences. However, short text is much more sparse, which becomes a bottleneck to achieving good accuracy (Li et al. 2016). Opinion mining is even more challenging (Pak and Paroubek 2010) since, among others, sarcasm is hard to detect by algorithms (Eke et al. 2020).

Our goal is to enhance Twitter content classification by involving information on the user interaction network. We combine text classifiers as baseline methods with several network classification methods (Nandanwar and Murty 2016). These methods learn to classify nodes using a manually labeled training set, usually based on the assumption that linked entities are related (Sen et al. 2008).

For network classification, an emerging class of methods is node embedding, which is the counterpart of the text embedding methods. To embed nodes in a vector space, we consider edge sequences from the network as sentences and apply text embedding methods (Perozzi et al. 2014). Given a vector representation of each node, standard classifiers can be used, similar to text classification based on word embedding.

Our final method combines text and network classifiers. While several methods exist to embed by text content (Tang et al. 2014) as well as by network structure (Rozemberczki et al. 2020), we are aware of only a few results that combine the two (Yang and Yang 2018; Zhuo et al. 2019; Gong et al. 2020).

**Fig. 1:**
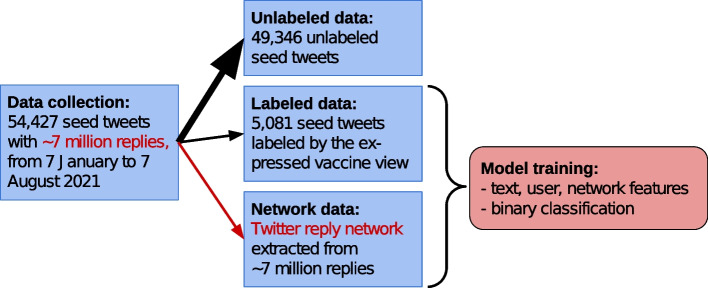
Overview of our data collection and experiments. In Fig. 10, the binary classification for vaccine skepticism detection is described in more detail

For our experiments, we compiled a data set from public COVID-19 vaccination-related tweets between 7 January 2021 and 7 August 2021, using the free Twitter API. We collected over 50,000 seed tweets that generated high activity and also collected the response to these tweets. An overview of our data collection and experiments are shown in Fig. 1. Similar experiments (Ng and Carley 2021) and a data set (Muric et al. 2021) were published very recently.

To evaluate our methods, we manually labeled over 10% of the seed tweets. We applied multiple data preprocessing steps, including language detection, to select English language content. Before our classification experiments, we also investigated the geographic distribution of our data collection and the share of anti-vaxxers in different regions.

Our main goal is to demonstrate the effectiveness of network embedding for classifying Twitter content as supporting pro- or anti-vaccination. Toward this end, we used a large variety of algorithms from the open-source Python library (Rozemberczki et al. 2020), including Laplacian Eigenmaps (Belkin and Niyogi 2001), Role2Vec (Ahmed et al. 2018), Diff2Vec (Rozemberczki and Sarkar 2018), Node2Vec (Grover and Leskovec 2016), DeepWalk (Perozzi et al. 2014), Walklets (Perozzi et al. 2016), and more.

In our evaluation, we ordered tweets by time and used the first 70% of the labeled data for training and the remaining 30% for evaluation. For a baseline, we used text classification by pre-trained BERT models (Devlin et al. 2018) combined with statistics of the past tweets of the same user. We selected the best-performing pre-trained model from the Huggingface library. In our main conclusion, using Walklets (Perozzi et al. 2016), we improve the AUC of anti-vaccination content detection from the baseline of 0.838 to 0.890. We also investigated how classification performance changed over time in our data collection period. We also evaluated the generalizability of our methods to out-of-domain vaccination content by training and testing across content in Europe and North America, which differ regarding the main concerns against vaccination.

The rest of this paper is organized as follows. After reviewing related results, in “Data” section, we describe the properties of data collection and labels. Finally, “Experiments” section describes and evaluates text and network embedding methods for classifying vaccination skeptic content.

## Related work

### Analysis of vaccine sentiment in social networks

The prime goal of social media analysis towards vaccination is the detection and isolation of anti-vaxxer communities (Mitra et al. 2016). By collecting social media content, one can assess the public opinion towards vaccination (Salathé and Khandelwal 2011) and even design communication to promote vaccination (Steffens et al. 2020). Recently, experiments to identify COVID-19 vaccine skeptic content (Ng and Carley 2021) and a data set (Muric et al. 2021) were also published. For the Central-Eastern-Europe region, where Facebook is the most popular social network platform, similar results appeared using Facebook data (Klimiuk et al. 2021); however, Facebook has no public data access API, so its availability is strongly limited for research. As another alternative platform, research using data from Reddit also has appeared (Melton et al. 2021). Finally, we mention that we conducted sentiment analysis on our Twitter collection based on geolocation and vaccine type in Béres et al. (2021a).

A few recent results are closely related to ours in that they also analyze Twitter data regarding COVID misinformation. In Cruickshank et al. (2021), sharing websites, a common communication strategy was analyzed, and the authors found even major news sites publishing unreliable information that, at the time of analysis, was unfounded, yet only a few websites expressed explicit opposition to vaccination. In Ginossar et al. (2022), the content and dynamics of YouTube videos shared in vaccine-related tweets posted to COVID-19 conversations before the COVID-19 vaccine rollout were investigated, most of which contained anti-vaccination frames based on conspiracy theories in a form of spamming and coordinated efforts. Finally, in Seo et al. (2022), it is experimentally proved that correction from the health organizations effectively reduced participants’ perceived accuracy rating on the COVID-19 fake news, which emphasizes the importance of detecting misinformation in social media.

### Vector space embedding for natural language processing

Embedding words of the human language into vector spaces by neural networks (Mikolov et al. 2013) revolutionized several natural language processing tasks, including text classification. As one such method, BERT (Devlin et al. 2018) is undoubtedly a breakthrough in the use of Machine Learning for Natural Language Processing. Its technological innovation lies in the bidirectional training of a Transformer, a popular attention model. The superior performance of BERT in several tasks is also attributed to its novel training procedure, where the model is jointly optimized for the masked language model and next-sentence prediction tasks. With just one additional output layer, a pre-trained BERT model can be easily fine-tuned for a selected downstream task such as Question Answering, Natural Language Inference, Named Entity Recognition and Text Classification.

Due to the hundreds of millions of model parameters, pre-training a BERT model has a high computational cost. To remedy this issue, several works (Sun et al. 2019; Turc et al. 2019; Tang et al. 2019) were proposed to compress or distill knowledge from large BERT models. For example, in Turc et al. (2019), a small BERT model with significantly fewer parameters is trained based on the predictions of a large pre-trained teacher model that resulted in comparable performance to the teacher.

The original BERT model was trained on BookCorpus, a dataset of unpublished books and English Wikipedia. Since then, several works have been published assessing models tailored for social network contexts, including emojis, hashtags or mentions. For example, Bertweet (Nguyen et al. 2020) is the first model pre-trained for English Tweets. Finally, in 2020 researchers published the first models (Nguyen et al. 2020; Müller et al. 2020) pre-trained on hundreds of millions of tweets related to COVID-19. Later in 2021, with the ongoing vaccination effort, a BERT model was developed for COVID vaccination-related fact classification.^1^

### Node embeddings

Node embedding methods form a class of network representation learning methods that map graph nodes to vectors in a low-dimensional vector space. They are designed to represent vertices with similar graph neighborhoods by vectors that are close in the vector space. Perhaps the most well-known method is Laplacian eigenmaps (Belkin and Niyogi 2001). Another class of models is based on the adjacency matrix of the graph; one popular example is graph factorization (Ahmed et al. 2013).

The research area of node embeddings has been recently catalyzed by deploying the idea of natural language embedding (Mikolov et al. 2013). Most node embedding methods sample random walks from the graph and use the sampled node sequences analogous to sentences in a large text document to learn an embedding as the representations of network nodes.

DeepWalk (Perozzi et al. 2014) was the first algorithm that extended the notion of word embeddings to network representation learning. However, DeepWalk representations use a method that turned out to be too rigid in controlling how node neighborhoods are explored. To solve this issue, Grover and Leskovec proposed Node2Vec (Grover and Leskovec 2016) by using parametrized random walks to find the best exploration strategy (BFS or DFS-like) for the task at hand. In another work (Perozzi et al. 2016), the authors reflected that DeepWalk is biased towards representations that preserve the lowest power of the adjacency matrix *A*, while in some applications, information on higher-order connectivity may offer performance benefits. Their algorithm, Walklets (Perozzi et al. 2016), solves this problem by subsequently learning representations from the graph at different scales. In contrast to GraRep Cao et al. (2015), where $A^k$ is explicitly factorized, Walklets only samples node pairs from the original random walks with different skipping factors *k*.

In recent years, random walk or diffusion-based approaches (Perozzi et al. 2014; Grover and Leskovec 2016; Ahmed et al. 2018; Perozzi et al. 2016; Rozemberczki and Sarkar 2018) were successfully applied for multi-label classification, link prediction, community detection and user deanonymization (Bères et al. 2021b) in various real-world networks. Usually, they significantly outperform classical approaches that are infeasible for large networks.

## Data

### Seed tweets

We collected a seed set of tweets that anyone can view using the free Twitter API. The collection spans the time period between 7 January 2021 and 7 August 2021. We specified our search query^2^ to collect tweets that received at least 50 replies within the first five days after publication; contain any of the seven vaccination-related keywords: “vaccine”, “vaccination”, “vaccinated”, “vaxxer”, “vaxxers”, “#CovidVaccine”, “covid denier”; but exclude the words “Trump”, “Biden”, “republican” and “democrat” to eliminate drift toward the US presidential inauguration in January 2021. By the above query, we collected 54,427 seed tweets. The three most popular languages in our collection are English (91.18 %), French (6.14 %) and Hindi (1.03 %).

### Twitter reply network

**Fig. 2:**
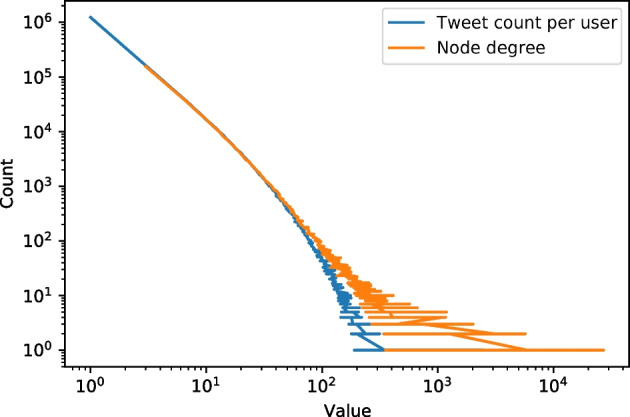
The user tweet count and node degree distributions in our Twitter reply network collection

We expanded our Twitter collection by gathering the replies to each seed tweet. By the extended collection, we are able to form a reply network between Twitter users. We define an undirected network such that there is an edge between user *u* and user *v* if *u* replied to a tweet of *v* (or visa versa) during the period of our data collection. Altogether, we collected 7.1 million replies; however, the majority of the users received only one reply in the data. For this reason, we reduced the reply network by dropping users with less than three connections. The final network has 579,159 nodes and 4,156,502 edges without multiplicity. In Fig. 2, we show the distribution of node degree and tweet number posted by the same user.

### Geographic distribution

**Fig. 3:**
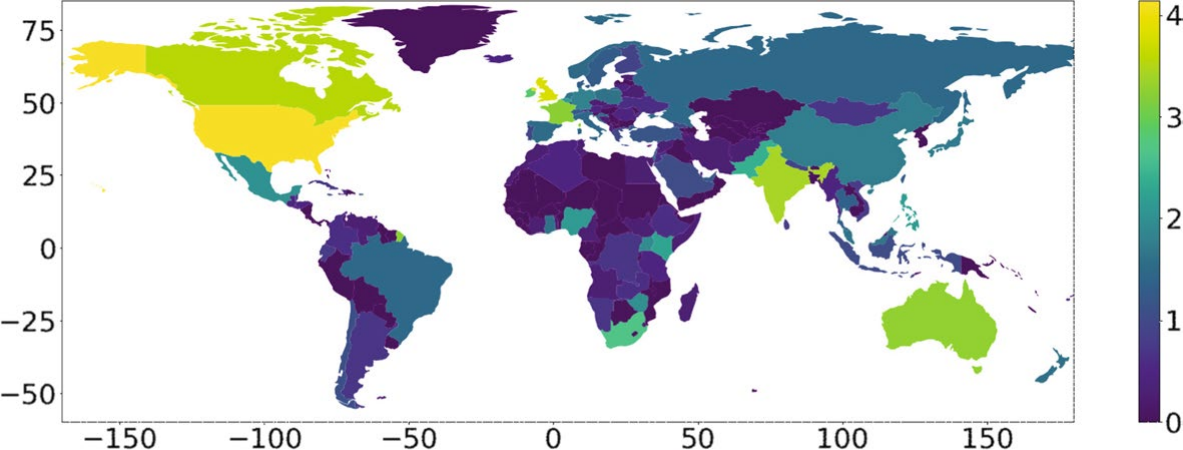
Geographic distribution of the seed tweets. Colors show the base 10 logarithm of the tweet count

**Fig. 4:**
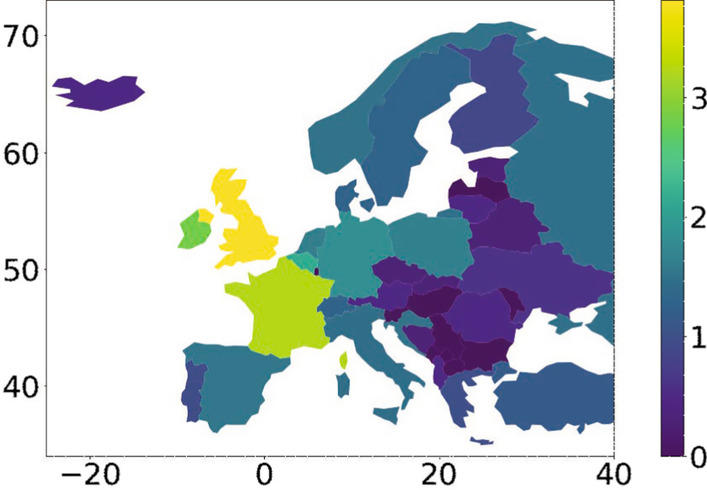
Geographic distribution of the seed tweets in Europe. Colors show the base 10 logarithm of the tweet count

The geographic distribution of the seed tweets in Figs. 3 and 4 aligns well with the general worldwide distribution of Twitter (Statista Research Department 2022; Humanitarian Data Exchange 2022), for example, low usage in Central Eastern Europe.

Since the location information of tweets is rather noisy, we applied the following data-cleaning procedure. For each tweet, we first extracted the location string (e.g., ”Washington, DC”, “London, UK”) from the posting user profile. If the location string was missing, we set the country based on the language of the tweet if it is spoken only in a single country (e.g., Italian). Then we assigned geographic coordinates to the extracted cities and countries by sending queries to Wikipedia.

The geographical analysis in Figs. 3 and 4 is based on 61% of the seed tweets. We excluded the remaining tweets from the visualization since they have missing, invalid (e.g., Around the world, Mars) or inconclusive (e.g., London-NYC-DC) location strings.

### Manual data annotation

**Fig. 5:**
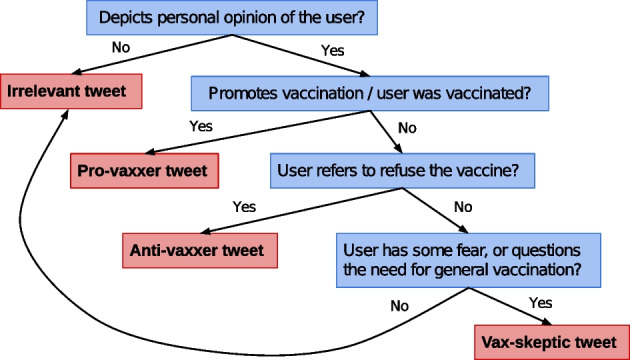
Definition for vaccine view categories that we used during the tweet labeling process. In Table 1, we show a few tweet examples along with their assigned label

**Fig. 6:**
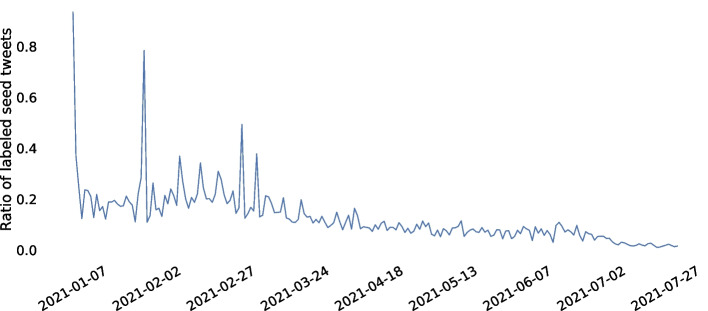
Daily ratio of labeled seed tweets

We annotated a random 11.45% of the English language seed tweets in our data set with four different labels: pro-vaxxer (2625 tweets), irrelevant (1436 tweets), vax-skeptic (676 tweets) and anti-vaxxer (344 tweets). The definition for each vaccine view category is described in Fig. 5, where we present a flowchart that guided our data labeling process. We found that it is even hard for humans to differentiate between vax-skeptic and anti-vaxxer content. Thus, we merged anti-vaxxers into the vax-skeptic category in our experiments. We started data annotation parallel with the data collection, which resulted in a decreasing labeled tweet ratio over time, as presented in Fig. 6. We note that due to limited resources for manual annotation, we did not attempt to analyze the accuracy and consistency of the labels by multiple labeling, and hence we cannot report inter-rater reliability.

**Table 1:** Example tweets for different vaccine view categories

Tweet example	Assigned label
@POTUS But Joe what if I lie and don’t have the vaccine and walk out without a mask?	Anti-vaxxer
Sobering. Met an old neighbour, known her for 11 years, sensible, bit of a rebel and free spirit. 16 months ago: Vaccine, no way! Now double jabbed, massive reaction to 1st, of course her kids are getting it and tearful indignation that I would not. From friends to enemy	Anti-vaxxer
So I am still waiting. Has ANYONE knowledge of a healthy baby/healthy mom delivered post COVID vaccine?	Vax-skeptic
Do you have family members pressuring you to get a COVID vaccine?	Vax-skeptic
I’m getting my 14yo vaccinated today. Trust the science. #GetVaccinated	Pro-Vaxxer
I have really had it with the anti-vaxxer crowd. You folks are as dangerous as guys with assault weapons.	Pro-Vaxxer
Fully vaccinated students do not need to wear masks in classrooms this fall, CDC says.	Irrelevant
Is it possible to develop some vaccine for Terrorism?	Irrelevant

**Fig. 7:**
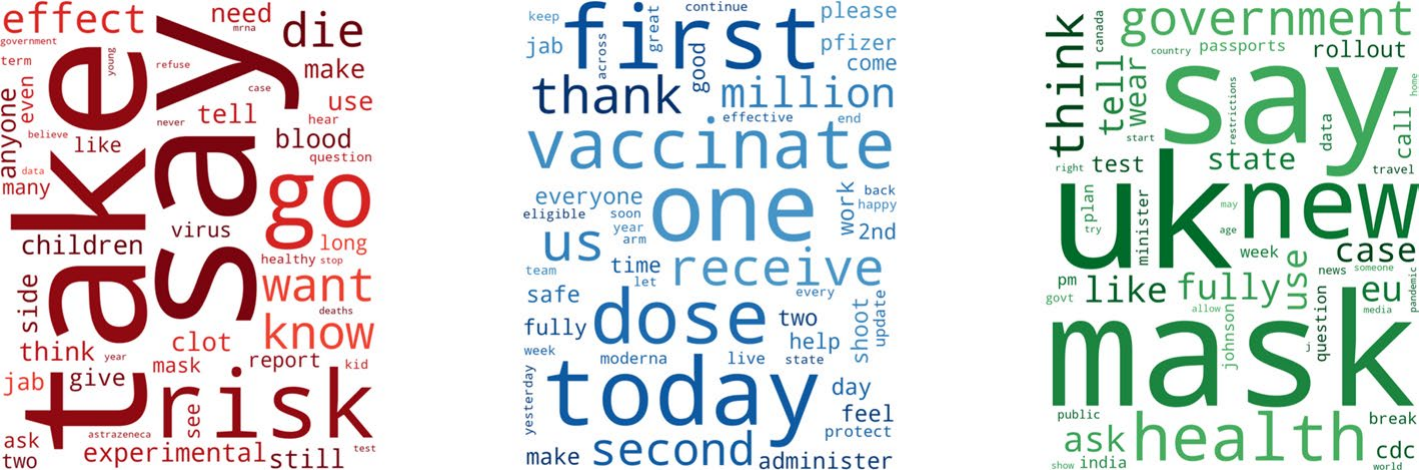
Popular words for tweets labeled as vax-skeptic (left), pro-vaxxer (center) and irrelevant (right)

The 50 most popular words for the three categories (pro-vaxxer, vax-skeptic and irrelevant) are shown in Fig. 7. Child vaccination, side effects, and death case reports were among the most popular topics for vax-skeptic users. Personal vaccination experience, daily administered vaccine status reports, and eligibility for different age groups was in the highlight for pro-vaxxers. Finally, in the irrelevant category, tweets mostly echo the official vaccine policy of governments, politicians or national health agencies. These tweets are usually relayed or posted by large news agencies that tend to have a neutral tone regarding vaccines.

**Fig. 8:**
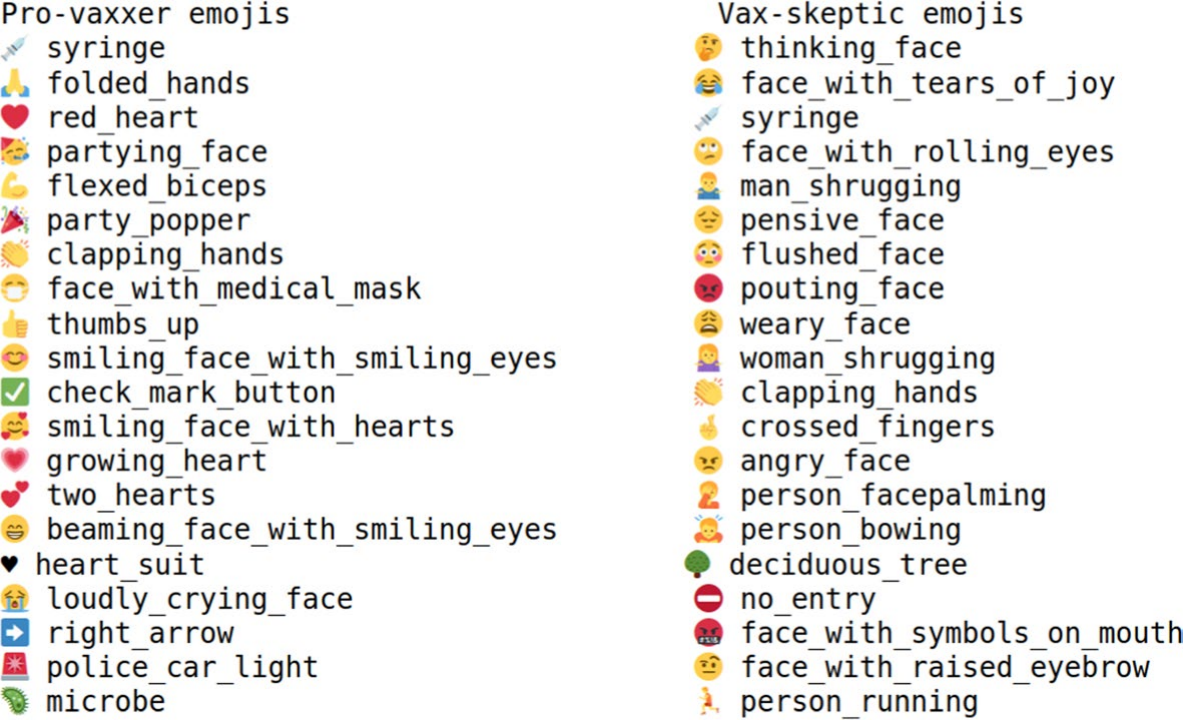
Popular emojis for pro-vaxxer (left) and vax-skeptic (right) tweets

A good separation in sentiment can also be observed for pro-vaxxer and vax-skeptic users through emojis. In Fig. 8, there are only two emojis (syringe, clapping hands) in the intersection for the 20 most popular emojis of pro-vaxxer and vax-skeptic tweets.

**Fig. 9:**
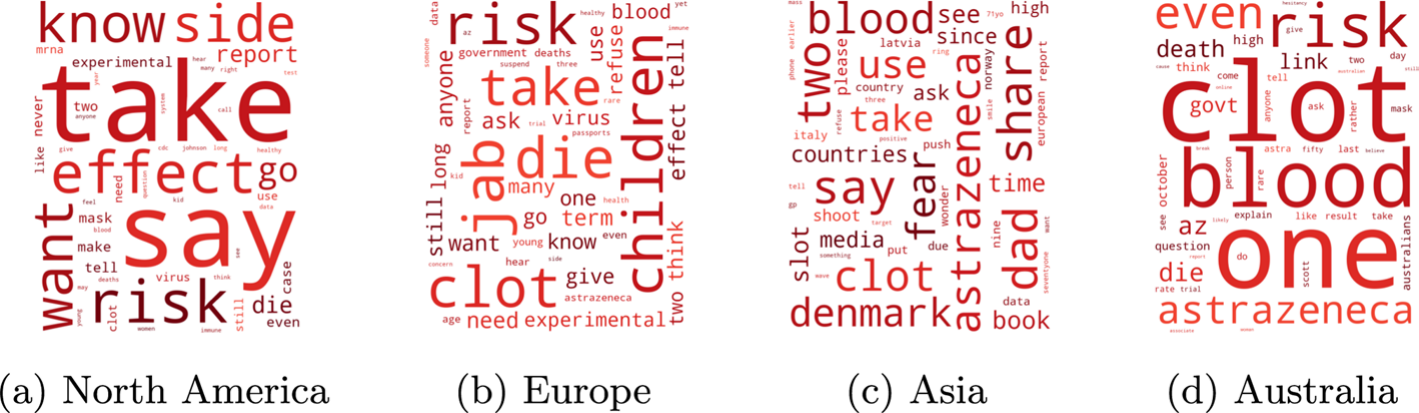
Popular words in vax-skeptic tweets in different continents

Finally, in Table 2, we report the geographic distribution of the seed tweets that we labeled pro-vaxxer or vax-skeptic. The dominance of English-speaking countries for each continent reflects that we only specified English keywords during the data collection process. It is interesting to see major differences in the vax-skeptic rate for different continents. To better understand this behavior, we visualize the 50 most popular words in vax-skeptic tweets for each continent in Fig. 9. These regions share various topics like possible side effects or the risk of vaccinating children, but the blood clot side effect cases of AstraZeneca have a significantly higher dominance in European and Australian tweets. Furthermore, tweets from these continents have a significantly higher vax-skeptic ratio see Table 2) than North America, likely due to the fact that the North American population was not widely vaccinated with AstraZeneca. Furthermore, in Europe and Australia, vax-skeptic users actively question the government’s vaccination policy, which might also explain the high vax-skeptic rates.

**Table 2:** Tweet count, vax-skeptic and pro-vaxxer rate by continents for labeled data

Labeled tweets					
Continent	Countries	Number of tweets:		Vax-skeptic ratio (%)	Pro-vaxxer ratio (%)
		By country	By continent		
North America	United States	1430	1757	15.65	58.96
	Canada	317			
	Other	10			
Europe	United Kingdom	909	1039	25.31	46.48
	Ireland	79			
	Other	51			
Asia	India	163	211	8.05	53.55
	Pakistan	24			
	Other	24			
Australia	Australia	116	116	19.82	31.03
Other	–	–	108	14.81	49.07
Missing	–	–	1850	23.02	48.86

## Experiments

### Evaluation setup

We consider two separate classification tasks for vaccination skepticism and support. To classify vaccine skepticism, we used pro-vaxxer and irrelevant as one class against vax-skeptic and anti-vaxxer as the other. The vax-skeptic and anti-vaxxer rate in the data used for classification is approximately 20% (1020 out of 5081 tweets). And for the other task, we classified pro-vaxxer against all other labels. The pro-vaxxer ratio is approximately 51% (2625 out of 5081 tweets).

For classification, we used the following attributes for each tweet:

1. *Text* First, we clean raw tweet text by removing URLs, mentions and the hashmark symbol from hashtags. Then, we transform emojis into textual descriptors with the Python emoji^3^ package. Finally, the cleaned tweet text is fed to a BERT model and we use its output as a 768-dimensional representation of the text.
2. *User history* Four basic statistics (minimum, maximum, mean, standard deviation) calculated for the past tweet labels of the same user to represent user vaccine view;
3. *Network statistics* Twitter user features such as the number of followers, friends, posts, and likes as well as some node centrality metrics (indegree, outdegree, core number, PageRank) calculated from the Twitter reply network that we extracted from the data.
4. *Raw network* 128-dimensional user representation obtained by Walklets Perozzi et al. (2016) over the Twitter reply network.

**Fig. 10:**
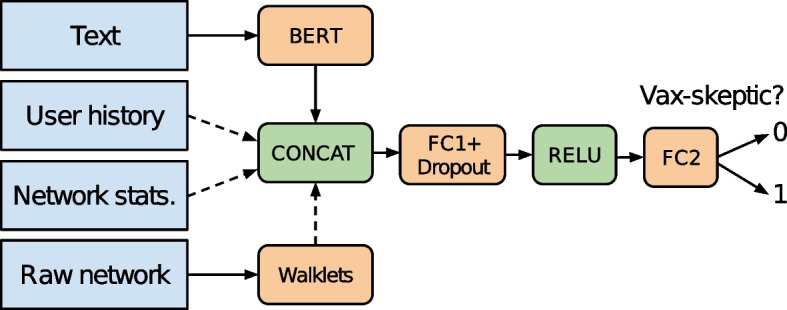
Schematic view of the pro- versus anti-vaxxer classifier framework. Tweet text and network representations are obtained by BERT and Walklets, respectively. User history, network statistics and raw network vectors can be independently turned on and off. The downstream classifier consists of two fully connected (FC1, FC2) layers

The vector representation from the four modalities is concatenated and fed through two fully connected layers (FC1: 768–1172 dimensional depending on enabled features, FC2: 128 dimensional), as seen in Fig. 10. We used dropout with a value of 0.1 for the first fully connected layer (FC1) to avoid overfitting. We note that user history, network statistics and raw network representations can be independently turned on and off in our experiments. Furthermore, both of these feature vectors are standard normalized before concatenation.

In our experiments, we split the tweet data along the time axis into a 70% training and a 30% testing sets, that is 3556 and 1525 tweets, respectively. We report the average performance of the binary classifier on the test tweet set in terms of area under the ROC curve (AUC) based on ten independent instances. Both the architecture and the parameters of our neural classifier were optimized for AUC on the test set.

### BERT model performance discussion

In our first experiments, we selected 11 pre-trained BERT models from Huggingface^4^ to measure the performance of the text-only approach. Table 3 shows the performance and query time for each selected BERT model. It is important to note that we did not execute any further training procedures for the selected models. We used the text embedding returned by BERT to classify each tweet with the downstream classifier of Fig. 10.

**Table 3:** Performance (AUC) and query time (seconds) of different BERT models for both vax-skeptic and pro-vaxxer content prediction

Model	Parent model	Original task	Published	Downloads	Vax-skeptic	Pro-vax	Time
					AUC	AUC	
Vaccinating-covid-tweets (Pak and Paroubek 2010)	Bertweet-base	Classification	2021	19 K	**0.810**	0.747	12.98
Bert-small (Bhargava et al. 2021; Turc et al. 2019)	–	Fill-Mask	2019	161 K	0.793	0.743	3.88
Covid-twitter-bert (Müller et al. 2020)	Bert-large	Fill-Mask	2020	8.5 K	0.787	**0.753**	34.72
Bert-medium (Bhargava et al. 2021; Turc et al. 2019)	–	Fill-Mask	2019	72.5 K	0.779	0.732	6.17
Bertweet-covid19-base (Nguyen et al. 2020)	Bertweet-base	Fill-Mask	2020	24.9 K	0.766	0.737	12.89
Bertweet-base (Nguyen et al. 2020)	Bert-base	Fill-Mask	2020	71.4 K	0.765	0.742	13.01
Bert-mini (Bhargava et al. 2021; Turc et al. 2019)	–	Fill-Mask	2019	54.5 K	0.751	0.701	2.66
Bert-tiny (Bhargava et al. 2021; Turc et al. 2019)	–	Fill-Mask	2019	129 K	0.709	0.639	1.84
Bert-base (Devlin et al. 2018)	–	Fill-Mask	2018	16107 K	0.709	0.700	13.53
Bertweet-large (Nguyen et al. 2020)	Bert-large	Fill-Mask	2020	8 K	0.575	0.605	34.05
Bert-large (Devlin et al. 2018)	–	Fill-Mask	2018	532 K	0.556	0.580	34.52

The best performance is marked by boldface for each task. Typically, models pre-trained on COVID-19-related tweets perform better, while large BERT models overfit. The number of monthly downloads on Huggingface shown in the fifth column was accessed on 11 April 2022

Our results show that models pre-trained on COVID-19-related tweets tend to perform better. One of the best-performing model^5^ (*vaccinating-covid-tweets*) was originally developed to classify English tweets whether they contain any facts about COVID-19 vaccines, the other^6^ (*covid-twitter-bert*) even before vaccines became available. It is also interesting that smaller BERT models not pre-trained for COVID-19-related data (*bert-small*, *bert-medium*) are not far behind in performance with a significantly faster running time. Thus, we see the further training of the *bert-small* architecture on COVID-19-related tweets as a prominent direction of future work. On the other hand, large general BERT models (*bert-large*, *bertweet-large*) overfit for this classification task. Finally, the least complex BERT architectures (*bert-mini*, *bert-tiny*) also have low performance.

### Performance with different feature sets

Table 4 shows the main results that we achieved by adding user history, network statistics and node embedding (raw network) vectors to the classifier in addition to the BERT representation of the tweet text. In these experiments, we only use the best-performing BERT model for each task, *vaccinating-covid-tweets* for vaccine skepticism and *covid-twitter-bert* for pro-vaccine content classification, respectively. Not surprisingly, statistics on past tweets of the same user have a strong contribution to the text-only approach, as users usually stick to their opinion. On the other hand, simple network statistics from Twitter have less effect on model performance.

**Table 4:** Model performance (AUC) for both vax-skeptic and pro-vaxxer content prediction with different feature sets (Text, User history, Network statistics, Raw network)

Text	User history	Network statistics	Raw network	AUC		T-test	
				Score	Gain (%)	t	p
(a) Vax-skeptic content prediction							
$\checkmark$				0.810	–	19.983	9.2e−22
$\checkmark$	$\checkmark$			0.840	3.7	9.645	9.2e−12
$\checkmark$		$\checkmark$		0.832	2.7	16.330	9.1e−19
$\checkmark$			$\checkmark$	0.886	9.3	–	–
$\checkmark$	$\checkmark$	$\checkmark$	$\checkmark$	0.887	9.5	−0.825	0.414
(b) Pro-vaxxer content prediction							
$\checkmark$				0.753	–	14.038	1.2e−16
$\checkmark$	$\checkmark$			0.786	4.4	6.313	2.1e−07
$\checkmark$		$\checkmark$		0.761	1.1	10.986	2.3e−13
$\checkmark$			$\checkmark$	0.812	7.8	–	–
$\checkmark$	$\checkmark$	$\checkmark$	$\checkmark$	0.816	8.3	−0.618	0.540

**Fig. 11:**
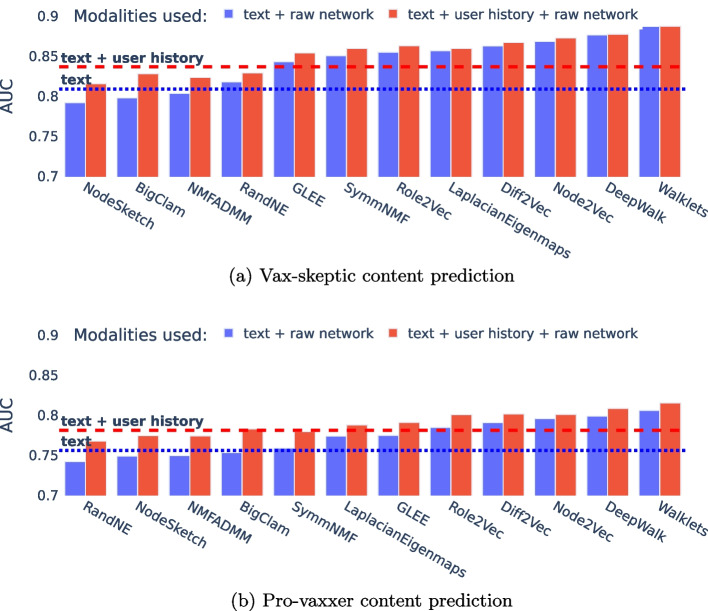
Performance (AUC) using different node embedding and community detection methods

The performance gain is shown over classification based on text only. Here, raw network represents Walklets (Perozzi et al. 2016), the best-performing node embedding model as seen in Fig. 11. Columns on the right report results of T-tests against text + raw network using 20 independent training-testing samples

Next, from the Karateclub Rozemberczki et al. (2020) Python library, we trained 12 node embedding and overlapping community detection algorithms that were able to process our Twitter reply network with more than 4 million nodes. In Fig. 11, we show that random walk or diffusion-based approaches [Walklets (Perozzi et al. 2016), DeepWalk (Perozzi et al. 2014), Node2Vec (Grover and Leskovec 2016), Diff2Vec (Rozemberczki and Sarkar 2018), Role2Vec (Ahmed et al. 2018)] perform better in this task than models based on matrix factorization (Sun and Févotte 2014; Kuang et al. 2012; Yang and Leskovec 2013) or spectral clustering (Belkin and Niyogi 2001; Torres et al. 2020).

**Fig. 12:**
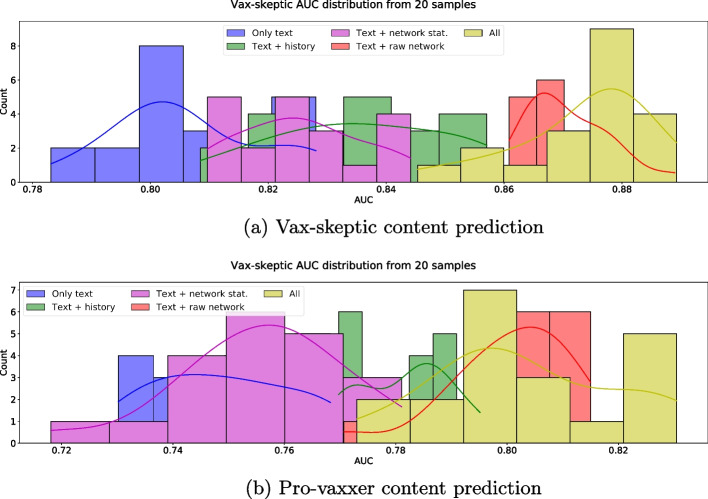
AUC distribution for different feature sets over the 20 independent data samples used for hypothesis testing

We investigate the statistical significance of the differences in AUC by generating 20 independent training-testing bootstrap samples by the scikit-learn resample function.^7^ Our experiments show that the mean AUC score for using *text + raw network* is significantly higher than that using only tweet text, user history or network statistics. We report the AUC distribution in Fig. 12 and the *t* and *p* values in Table 4.

**Fig. 13:**
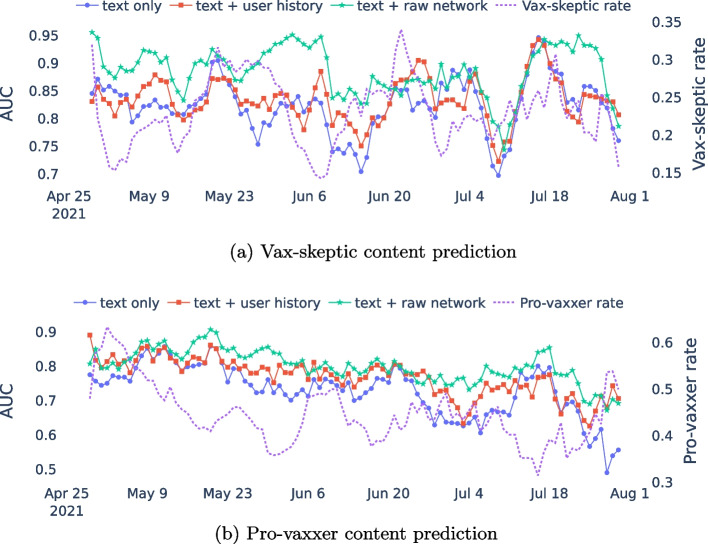
Model performance (AUC) and vax-skeptic/pro-vaxxer ratio based on a 7-day sliding window in the testing set

Finally, we analyze the performance of each feature set over time in Fig. 13. We calculate the AUC and the vax-skeptic/pro-vaxxer tweet ratio for a seven-day sliding window. Despite the major changes in the vax-skeptic rate, by using Walklets (Perozzi et al. 2016), the classifier can maintain its superior performance compared to using only text and historical vaccine view vectors. For example, in Fig. 13a, there is a sudden performance drop for the text-only (blue) and text with user history (red) settings around 18 July 2021, while the high performance is maintained by combining text and network embedding (green), even though these are part of the latest tweets in the testing set.

### Out of context evaluation

While our data collection includes only COVID vaccination tweets, we attempted to validate the generalizability of our methods to general content on anti-vaccination and vaccine skepticism, a topic much older than COVID. Our experiment begins with the observation in Fig. 9 that Europe and North America strongly differ in anti-vaccination content, in particular in that AstraZeneca blood clot side effect appears as a major concern in Europe and does not appear in North America at the time of our collection.

The evaluation results of training and testing in combinations of European and North American content are given in Tables 5 and 6. Our results in Tables 5d and 6d show that models using Walklets (Perozzi et al. 2016) vectors generalize extremely well across different regions by significantly improving the text-only approach. In Table 7, we report the statistical significance of the differences by using 20 bootstrap samples, as described in “Performance with different feature sets” section. In all cases, the use of the raw network significantly improves the AUC over other feature combinations.

**Table 5:** Cross-region model evaluation for vax-skeptic content prediction with respect to different modalities

	EU	US	Diff (%)
(a) Only text			
EU	0.775	0.771	0.5
US	0.711	0.745	4.7
(b) Text + user history			
EU	0.836	0.826	1.2
US	0.783	0.811	3.6
(c) Text + network stats			
EU	0.842	0.780	7.9
US	0.717	0.769	7.2
(d) Text + raw network			
EU	0.885	0.874	1.2
US	0.803	0.846	5.3
(e) All			
EU	0.888	0.876	1.4
US	0.832	0.847	1.8

In each table marked by letters (a–e), columns and rows represent the training and test regions, respectively. Here, raw network represents Walklets (Perozzi et al. 2016), the best-performing node embedding model (see Fig. 11). The *diff* column shows the AUC difference between training and testing on the same domain versus out-of-domain

**Table 6:** Cross-region model evaluation for pro-vaxxer content prediction with respect to different modalities

	EU	US	Diff (%)
(a) Only text			
EU	0.735	0.779	−5.9
US	0.687	0.721	4.9
(b) Text + user history			
EU	0.764	0.806	−5.5
US	0.728	0.762	4.6
(c) Text + network stats			
EU	0.774	0.773	0.1
US	0.680	0.732	7.6
(d) Text + raw network			
EU	0.829	0.843	−1.7
US	0.757	0.792	4.6
(e) All			
EU	0.829	0.837	−0.9
US	0.762	0.797	4.5

In each table marked by letters (a–e), columns and rows represent the training and test regions, respectively. Here, raw network represents Walklets (Perozzi et al. 2016), the best-performing node embedding model (see Fig. 11). The *diff* column shows the AUC difference between training and testing on the same domain versus out-of-domain

**Table 7:** T-test results of AUC values based on 20 bootstrap samples against the *text + raw network* modality

Modalities used	EU $\rightarrow$ EU		EU $\rightarrow$ US		US $\rightarrow$ EU		US $\rightarrow$ US	
	t	p	t	p	t	p	t	p
(a) Vax-skeptic content prediction								
Only text	16.3	1.1e−18	6.4	1.4e−07	11.7	4.1e−14	10.1	2.3e−12
Text + history	6.7	6.5e−08	0.5	6.1e−01	5.9	8.8e−07	4.7	3.9e−05
Text + network stat.	4.4	8.0e−05	7.9	1.5e−09	11.7	3.6e−14	6.3	2.4e−07
(b) Pro-vaxxer content prediction								
Only text	11.0	2.1e−13	5.9	8.2e−07	6.0	5.0e−07	7.1	1.9e−08
Text + history	7.1	1.7e−08	3.0	4.3e−03	5.5	2.4e−06	5.0	1.2e−05
Text + network stat.	5.9	7.3e−07	7.9	1.8e−09	7.9	1.3e−09	7.1	2.0e−08

Columns represent the training and testing region combinations, e.g. EU $\rightarrow$ US for training on EU and testing on US data. All differences are statistically significant

When comparing between training and testing on the same domain versus out-of-domain, training on the same domain is better, especially in the case of vax-skeptic classification (Table 5) and pro-vaxxer in the US. Pro-vaxxer classification AUC in the EU is very similar whether trained by EU or US training data; sometimes, out-of-context is even better (negative differences in Table 6. This indicates that pro-vaxxer content is similar, but vax-skeptic is different in the EU versus US, for example, regarding the AstraZeneca blood clot side effect issue. The smallest AUC difference between training in the same or out-of-domain is measured when using all modalities for vax skeptic classification, which shows the transferability of network embedding between the regions. Overall, our experiments suggest that the data set might support the generalization of identifying anti-vaccination content beyond a given context.

### Relation of node embedding to content semantics

**Fig. 14:**
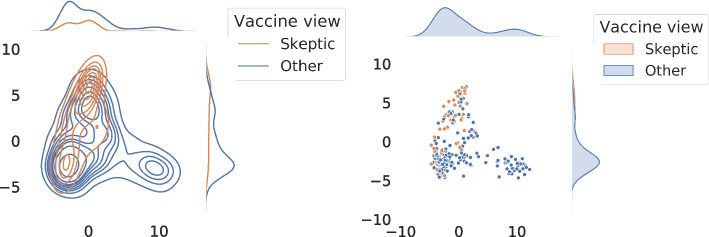
Walklets representation of the users mapped to a two dimensional vector space using PCA. Colors indicate users with different vaccine views. Left: kernel density estimation of vax-skeptic users compared to other accounts over the whole test period. Right: coordinates of the users active between 5–13 May

To confirm that network embedding alone is capable of capturing the semantics of the messages, we make an attempt to visualize and interpret the 128-dimensional embedding space of Walklets Perozzi et al. (2016), the best-performing node embedding algorithm in our experiments. Towards this end, first, we use PCA to map Walklets user representations into a two-dimensional vector space for visualization. In Fig. 14, our results show that there is indeed an observable separation between pro-vaxxer and vax-skeptic users.

Next, we explore the topic hierarchy of vax-skeptic and pro-vaxxer users in the Walklets embedding space. We select frequently appearing relevant terms such as blood clot manually from the word clouds, for example, in Figs. 7 and 9. For a term, we assign the average coordinate of the users who used the term in their tweets. Again, we visualize in two dimensions, but this time we deploy PCA for dimensionality reduction. We reduce the Walklets embedding space to 2 dimensions independently for the skeptic and pro-vaxxer users by applying PCA separately for the two subsets of users.

**Fig. 15:**
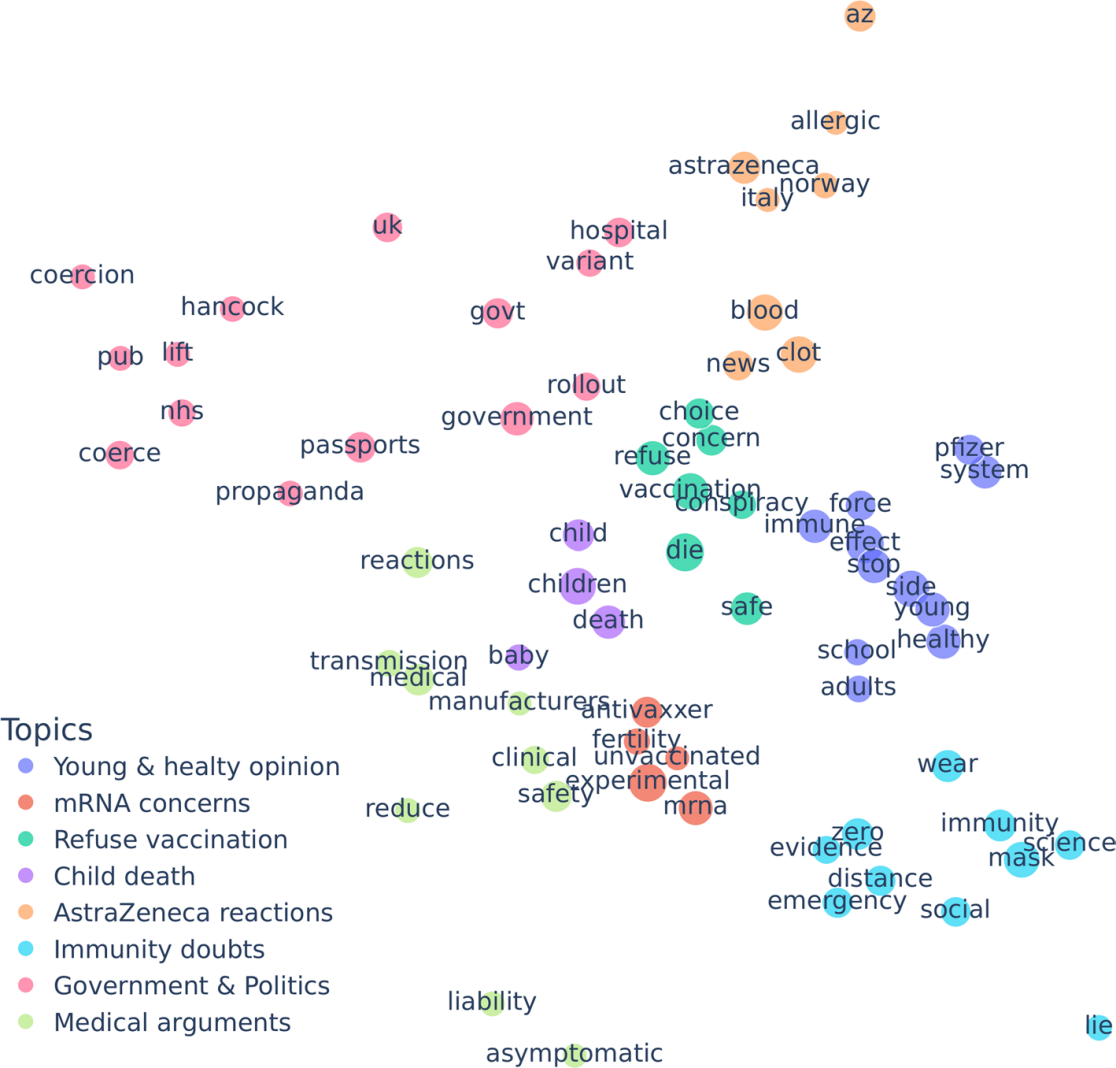
Vax-skeptic topic space uncovered by Walklets embeddings

First, we analyze the 2D plot of the manually selected vax-skeptic terms in Fig. 15. In the center, there are three anti-vaxxer topics related to child death, fear from the mRNA-based technology, and vaccine refusal induced by safety or free choice concerns. The periphery includes less offensive topics like politics, medical arguments, discussions related to AstraZeneca reactions, immunity doubts, and whether young and healthy people should generally vaccinate or not.

**Fig. 16:**
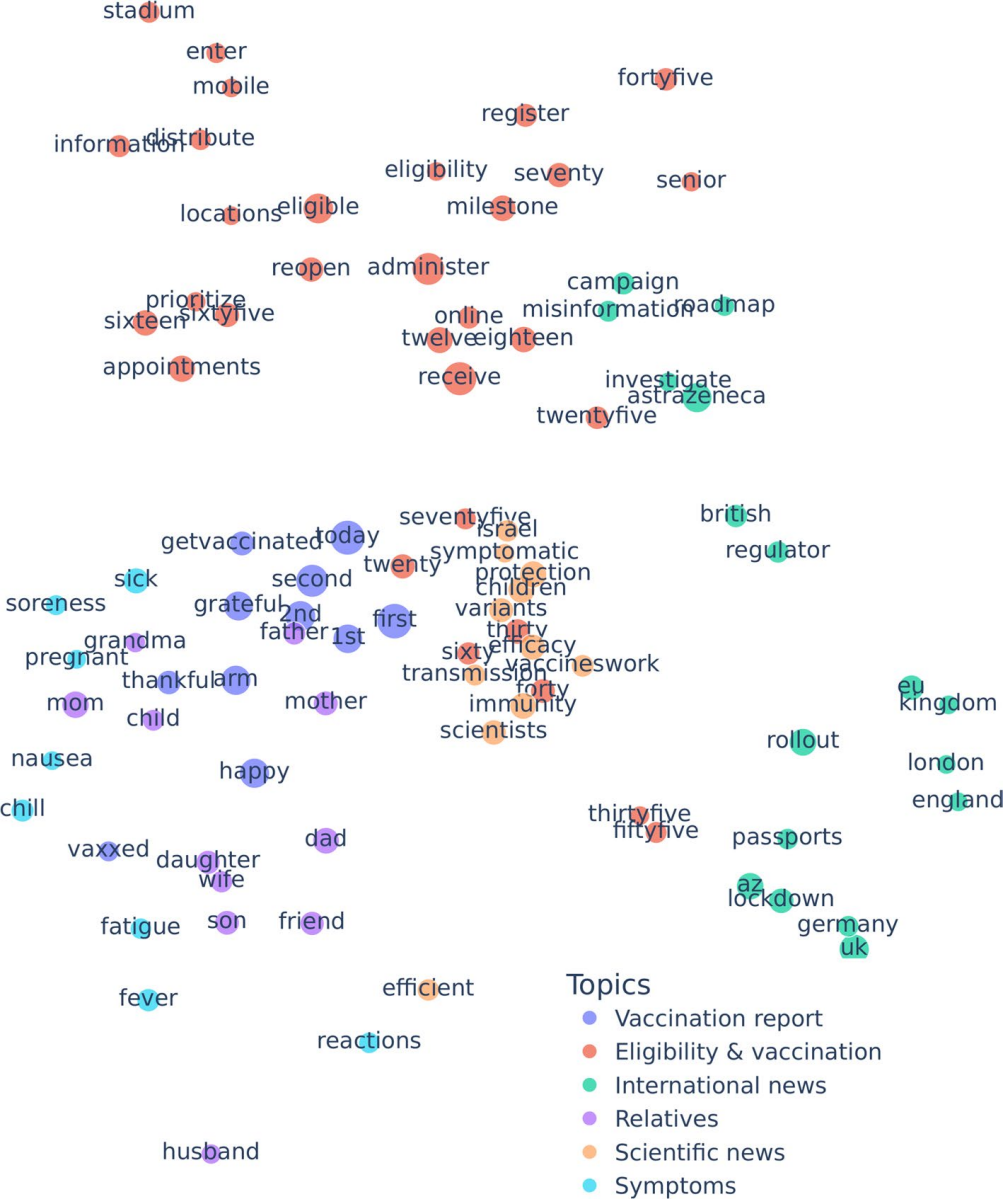
Pro-vaxxer topic space uncovered by Walklets embeddings

Next, we analyze pro-vaxxer terms in Fig. 16. Scientific news related to different levels of protection offered by COVID-19 vaccines is at the center. Two adjacent topics in the top-right region of the representation space are international news and the vaccination process in general, including eligibility for different age groups. Finally, in the bottom-left region, we can see personal vaccination reports, sometimes related to friends and family. A few side effects and post-vaccination symptoms are closely related to these conversations.

## Conclusion

In this work, we quantitatively showed that social interactions play a major role in detecting vaccine skepticism by analyzing our Twitter collection that spans the first seven months of 2021. We collected over 50,000 seed tweets of high engagement and relevance to COVID-19 vaccination and formed a reply network of users by adding edges between two users if one of them replied to the other user’s tweet. By deploying multiple node embedding models over the reply network, we improved the AUC of classifying pro- and anti-vaxxers by. Our baseline classifier relied on the BERT embedding vectors of tweet text and statistics on past tweets of the same user as features. In addition, we managed to discover pro-vaxxer and vax-skeptic communities by using the vector space embedding of nodes by Walklets, which successfully captured the topic hierarchy for different vaccine views on a very fine-grained level.

For reproducibility and future research purposes, we share our data on GitHub.^8^ In order to comply with the data publication policy of Twitter, we only share the user ID, original and reply tweet IDs along with the encoded content vectors.^9^

## Data Availability

The collected data and software code are publicly available in the GitHub repository https://github.com/ferencberes/covid-vaccine-network.

## Footnotes

1 https://huggingface.co/ans/vaccinating-covid-tweets.

2 Our search query executed at 7/20/21 12:34 AM: (vaccine OR vaccination OR vaccinated OR vaxxer OR vaxxers OR #CovidVaccine OR ”covid denier”) -democrat -republican -trump -biden min_replies:50 until:2021-07-16 since:2021-07-15.

3 https://pypi.org/project/emoji/.

4 https://huggingface.co/models.

5 https://huggingface.co/ans/vaccinating-covid-tweets.

6 https://huggingface.co/digitalepidemiologylab/covid-twitter-bert.

7 https://scikit-learn.org/stable/modules/generated/sklearn.utils.resample.html.

8 https://github.com/ferencberes/covid-vaccine-network.

9 https://huggingface.co/ans/vaccinating-covid-tweets.

## References

[CR1] Ahmed A, Shervashidze N, Narayanamurthy S, Josifovski V, Smola AJ (2013) Distributed large-scale natural graph factorization. In: Proceedings of the 22nd international conference on World Wide Web. WWW ’13. Association for Computing Machinery, New York, pp 37–48. 10.1145/2488388.2488393

[CR2] Ahmed N, Rossi R, Lee J, Kong X, Willke T, Zhou R, Eldardiry H (2018) Learning role-based graph embeddings. In: StarAI Workshop, IJCAI 2018, pp 1–8

[CR3] Ball P (2020). Anti-vaccine movement could undermine efforts to end coronavirus pandemic, researchers warn. Nature.

[CR4] Belkin M, Niyogi P, Dietterich T, Becker S, Ghahramani Z (2001). Laplacian eigenmaps and spectral techniques for embedding and clustering. Advances in neural information processing systems.

[CR5] Béres F, Csoma R, Michaletzky T, Benczúr A (2021a) Covid vaccine sentiment dashboard based on twitter data. Sci Secur 2(4):418–427

[CR6] Bères F, Seres IA, Benczúr AA, Quintyne-Collins M (2021b) Blockchain is watching you: profiling and deanonymizing ethereum users. In: 2021 IEEE international conference on decentralized applications and infrastructures (DAPPS), pp 69–78. 10.1109/DAPPS52256.2021.00013

[CR7] Bhargava P, Drozd A, Rogers A (2021) Generalization in NLI: ways (not) to go beyond simple heuristics. arXiv:2110.01518

[CR8] Brown T, Mann B, Ryder N, Subbiah M, Kaplan JD, Dhariwal P, Neelakantan A, Shyam P, Sastry G, Askell A (2020). Language models are few-shot learners. Adv Neural Inf Process Syst.

[CR9] Cao S, Lu W, Xu Q (2015) Grarep: learning graph representations with global structural information. In: Proceedings of the 24th ACM international on conference on information and knowledge management. CIKM ’15. Association for Computing Machinery, New York, pp 891–900. 10.1145/2806416.2806512

[CR10] Cruickshank I, Ginossar T, Sulskis J, Zheleva E, Berger-Wolf T (2021). Content and dynamics of websites shared over vaccine-related tweets in covid-19 conversations: computational analysis. J Med Internet Res.

[CR11] Devlin J, Chang M, Lee K, Toutanova K (2018) BERT: pre-training of deep bidirectional transformers for language understanding. CoRR. arXiv:1810.04805

[CR12] Eke CI, Norman AA, Shuib L, Nweke HF (2020). Sarcasm identification in textual data: systematic review, research challenges and open directions. Artif Intell Rev.

[CR13] Eysenbach G, Powell J, Kuss O, Sa E-R (2002). Empirical studies assessing the quality of health information for consumers on the world wide web: a systematic review. JAMA.

[CR14] Ginossar T, Cruickshank IJ, Zheleva E, Sulskis J, Berger-Wolf T (2022). Cross-platform spread: vaccine-related content, sources, and conspiracy theories in Youtube videos shared in early twitter covid-19 conversations. Hum Vaccines Immunother.

[CR15] Gong M, Yao C, Xie Y, Xu M (2020). Semi-supervised network embedding with text information. Pattern Recogn.

[CR16] Grover A, Leskovec J (2016) node2vec: scalable feature learning for networks. In: Proceedings of the 22nd ACM SIGKDD international conference on knowledge discovery and data mining 10.1145/2939672.2939754 PMC5108654 27853626

[CR17] Humanitarian Data Exchange (2022) Covid-19 Twitter data geographic distribution. https://data.humdata.org/dataset/covid-19-twitter-data-geographic-distribution. Accessed 21 March 2022

[CR18] Klimiuk K, Czoska A, Biernacka K, Balwicki Ł (2021). Vaccine misinformation on social media-topic-based content and sentiment analysis of polish vaccine-deniers’ comments on facebook. Hum Vaccines Immunother.

[CR19] Kuang D, Ding C, Park H (2012) Symmetric nonnegative matrix factorization for graph clustering, pp 106–117. 10.1137/1.9781611972825.10

[CR20] Li C, Wang H, Zhang Z, Sun A, Ma Z (2016) Topic modeling for short texts with auxiliary word embeddings. In: Proceedings of the 39th international ACM SIGIR conference on research and development in information retrieval, pp 165–174

[CR21] McMullan RD, Berle D, Arnáez S, Starcevic V (2019). The relationships between health anxiety, online health information seeking, and cyberchondria: systematic review and meta-analysis. J Affect Disord.

[CR22] Melton CA, Olusanya OA, Ammar N, Shaban-Nejad A (2021). Public sentiment analysis and topic modeling regarding covid-19 vaccines on the reddit social media platform: a call to action for strengthening vaccine confidence. J Infect Public Health.

[CR23] Mikolov T, Sutskever I, Chen K, Corrado G, Dean J (2013) Distributed representations of words and phrases and their compositionality. In: Proceedings of the 26th International conference on neural information processing systems—volume 2. NIPS’13. Curran Associates Inc., Red Hook, pp 3111–3119

[CR24] Mitra T, Counts S, Pennebaker JW (2016) Understanding anti-vaccination attitudes in social media. In: Tenth international AAAI conference on web and social media

[CR25] Müller M, Salathé M, Kummervold PE (2020) Covid-twitter-bert: a natural language processing model to analyse COVID-19 content on twitter. CoRR. arXiv:2005.07503 10.3389/frai.2023.1023281 PMC10043293 36998290

[CR26] Muric G, Wu Y, Ferrara E (2021) COVID-19 vaccine hesitancy on social media: building a public twitter dataset of anti-vaccine content, vaccine misinformation and conspiracies. CoRR. arXiv:2105.05134 10.2196/30642 PMC8694238 34653016

[CR27] Nandanwar S, Murty MN (2016) Structural neighborhood based classification of nodes in a network. In: Proceedings of the 22nd ACM SIGKDD international conference on knowledge discovery and data mining, pp 1085–1094

[CR28] Ng LHX, Carley K (2021) Flipping stance: social influence on bot’s and non bot’s covid vaccine stance. arXiv:2106.11076

[CR29] Nguyen DQ, Vu T, Nguyen AT (2020) BERTweet: a pre-trained language model for English Tweets. In: Proceedings of the 2020 conference on empirical methods in natural language processing: system demonstrations, pp 9–14

[CR30] Pak A, Paroubek P (2010) Twitter as a corpus for sentiment analysis and opinion mining. In: Proceedings of the seventh international conference on language resources and evaluation (LREC’10)

[CR31] Perozzi B, Al-Rfou R, Skiena S (2014) Deepwalk: online learning of social representations. In: Proceedings of the 20th ACM SIGKDD international conference on knowledge discovery and data mining. KDD ’14. ACM, New York, pp 701–710. 10.1145/2623330.2623732

[CR32] Perozzi B, Kulkarni V, Skiena S (2016) Walklets: multiscale graph embeddings for interpretable network classification. CoRR. arXiv:1605.02115

[CR33] Rozemberczki B, Sarkar R (2018) Fast sequence based embedding with diffusion graphs. In: International conference on complex networks, pp 99–107

[CR34] Rozemberczki B, Kiss O, Sarkar R (2020) Karate Club: an API oriented open-source python framework for unsupervised learning on graphs. In: Proceedings of CIKM. ACM, pp 3125–3132

[CR35] Salathé M, Khandelwal S (2011). Assessing vaccination sentiments with online social media: implications for infectious disease dynamics and control. PLoS Comput Biol.

[CR36] Sebastiani F (2002). Machine learning in automated text categorization. ACM Comput Surv (CSUR).

[CR37] Sen P, Namata G, Bilgic M, Getoor L, Galligher B, Eliassi-Rad T (2008). Collective classification in network data. AI Mag.

[CR38] Seo H, Xiong A, Lee S, Lee D (2022) If you have a reliable source, say something: effects of correction comments on covid-19 misinformation. In: Proceedings of the international AAAI conference on web and social media, vol 16, pp 896–907

[CR39] Statista Research Department (2022) Number of active Twitter users in selected countries. https://www.statista.com/statistics/242606/number-of-active-twitter-users-in-selected-countries. Accessed 21 March 2022

[CR40] Steffens MS, Dunn AG, Leask J, Wiley KE (2020). Using social media for vaccination promotion: practices and challenges. Digit Health.

[CR41] Sun DL, Févotte C (2014) Alternating direction method of multipliers for non-negative matrix factorization with the beta-divergence. In: 2014 IEEE international conference on acoustics, speech and signal processing (ICASSP), pp 6201–6205

[CR42] Sun S, Cheng Y, Gan Z, Liu J (2019) Patient knowledge distillation for bert model compression. arXiv:1908.09355

[CR43] Tang D, Wei F, Yang N, Zhou M, Liu T, Qin B (2014) Learning sentiment-specific word embedding for twitter sentiment classification. In: Proceedings of ACL, pp 1555–1565

[CR44] Tang R, Lu Y, Liu L, Mou L, Vechtomova O, Lin J (2019) Distilling task-specific knowledge from bert into simple neural networks. arXiv:1903.12136

[CR45] Torres L, Chan KS, Eliassi-Rad T (2020). GLEE: geometric Laplacian eigenmap embedding. J Complex Netw.

[CR46] Turc I, Chang M, Lee K, Toutanova K (2019) Well-read students learn better: the impact of student initialization on knowledge distillation. CoRR. arXiv:1908.08962

[CR47] Yang J, Leskovec J (2013) Overlapping community detection at scale: a nonnegative matrix factorization approach. In: Proceedings of the sixth ACM international conference on web search and data mining. WSDM ’13. Association for Computing Machinery, New York, pp 587–596. 10.1145/2433396.2433471

[CR48] Yang S, Yang B (2018) Enhanced network embedding with text information. In: 2018 24th international conference on pattern recognition (ICPR). IEEE, pp 326–331

[CR49] Zhuo W, Zhan Q, Liu Y, Xie Z, Lu J (2019). Context attention heterogeneous network embedding. Comput Intell Neurosci.

